# Unusual metastasis after radical cystectomy: case report

**DOI:** 10.1093/jscr/rjae112

**Published:** 2024-03-06

**Authors:** Mohammed H Mohammed, Fadel Mardnly, Mohamad Ghrer, Louei Alia, Lina W Assad

**Affiliations:** Urology, Damascus University, Al Assad University Hospital, Damascus 30621, Syria; Urology, Damascus University, Al Assad University Hospital, Damascus 30621, Syria; Urology, Damascus University, Al Assad University Hospital, Damascus 30621, Syria; Urology, Damascus University, Al Assad University Hospital, Damascus 30621, Syria; Pathology and Cytopathology, Damascus, Syria

**Keywords:** cheek metastasis, urothelial carcinoma, radical cystectomy

## Abstract

Cutaneous metastasis of urothelial carcinoma after radical cystectomy is extremely rare. We present the case of a 57-year-old man who underwent a radical cystectomy with ileal conduit for the presence of a bladder tumor. He developed a cheek lesion after 2 months, which was diagnosed as a metastatic nodule along with bone metastases from high-grade bladder urothelial carcinoma. This nodule was treated with surgical removal with subsequent chemotherapy, but he succumbed after 10 months due to widespread metastatic disease.

## Introduction

Bladder cancer is the 12th most common cancer worldwide with a high morbidity and mortality rate [1, 2]. Urothelial carcinoma (UC) is the most common type of bladder cancer. UC can also involve the renal pelvis and ureters, namely upper tract urothelial carcinoma (UTUC), and urethra [3]. At presentation, ~70% of bladder cancers are classified as non-muscle invasive (NMIBC); ~25% are muscle-invasive (MIBC); in the remaining 5% of cases, the cancer has already spread to the nearby tissues, lymph nodes, or to distant metastatic sites such as lungs and bones. Although only 5% of patients are metastatic at presentation, nearly 50% of patients with MIBC, undergoing curative-intent treatment, will eventually relapse and develop metastatic disease [4]. Radical cystectomy with extended pelvic lymph node dissection is considered the standard treatment for localized muscle invasive bladder cancer and recurrent high-grade noninvasive disease [5]. The reported incidence of cutaneous spread from primary urologic malignancies is 1.3%. Urinary bladder malignancies altogether account for 0.84% of cutaneous metastases [6]. Skin metastasis can present as nodular, inflammatory, and fibrotic type [6]. Nodular metastasis is common and may be of solitary or multiple type [7]. Here, we report a case of metastasis to the cheek from a UC of the bladder diagnosed and treated by radical cystectomy and chemotherapy before.

## Case report

**Figure 1:**
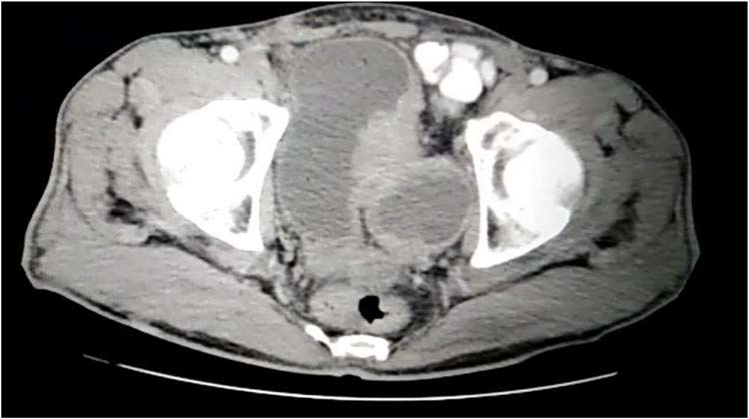
CT showing a large bladder diverticulum on the posterior wall with a mass within it.

**Figure 2:**
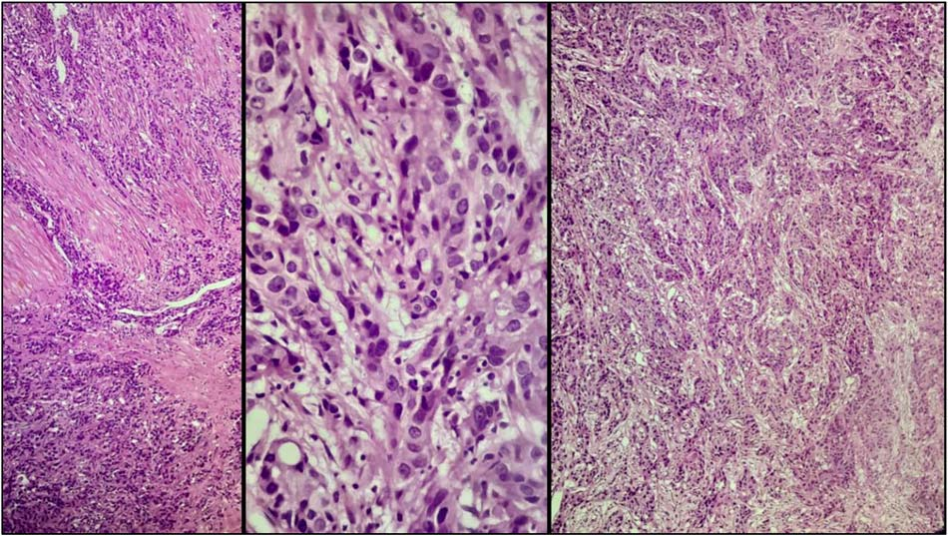
Microscopic examination showing neoplastic cells arranged in irregular nests invading the lamina propria and muscularis propria. Tumors cells showing nuclear pleomorphism, hyperchromasia, high N/C ratio with frequent mitotic figures.

**Figure 3:**
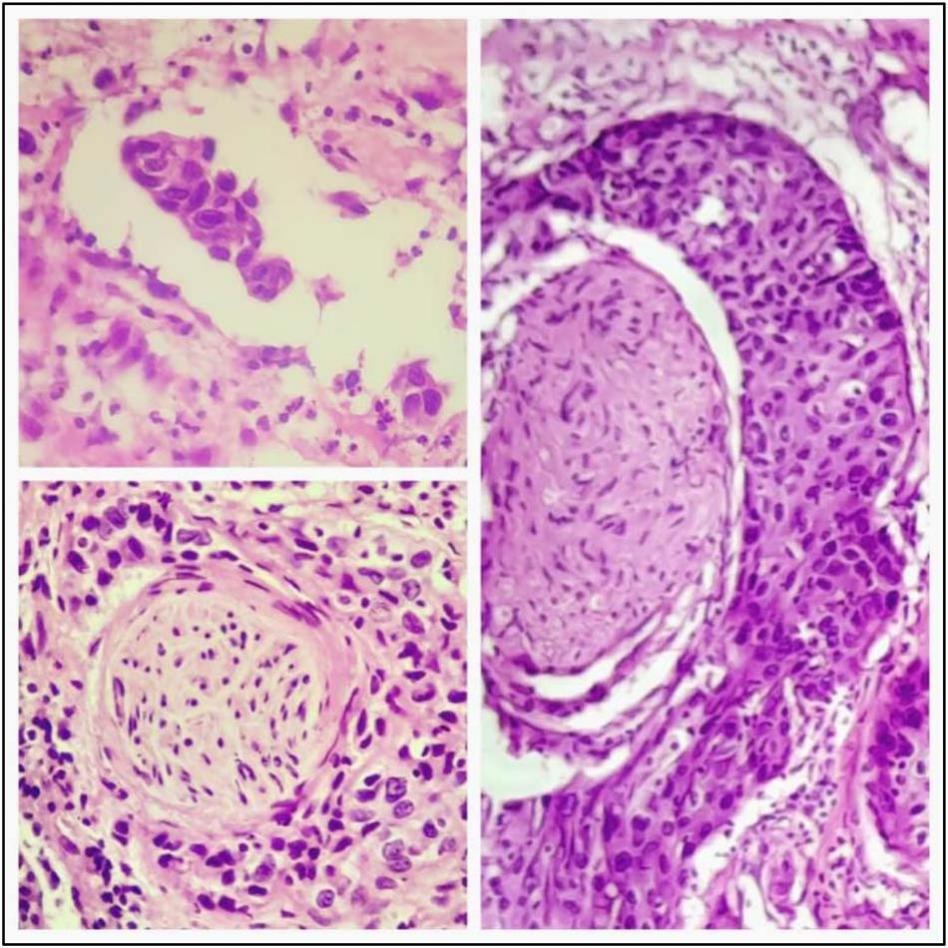
Lymphovascular and neural invasion is seen.

**Figure 4:**
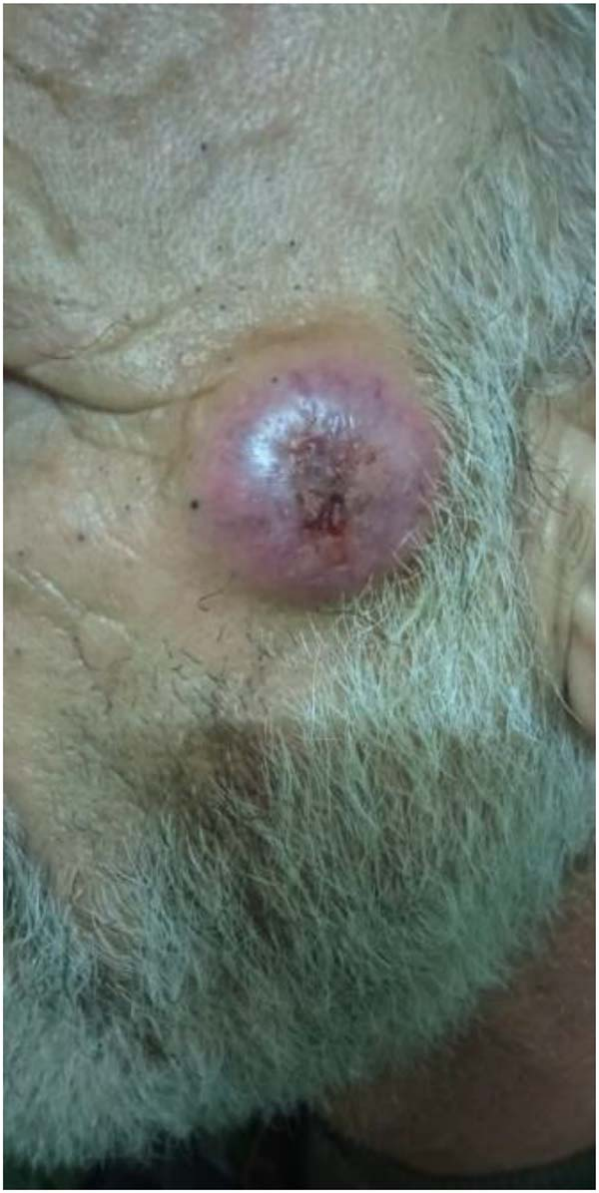
Metastatic at left cheek.

**Figure 5:**
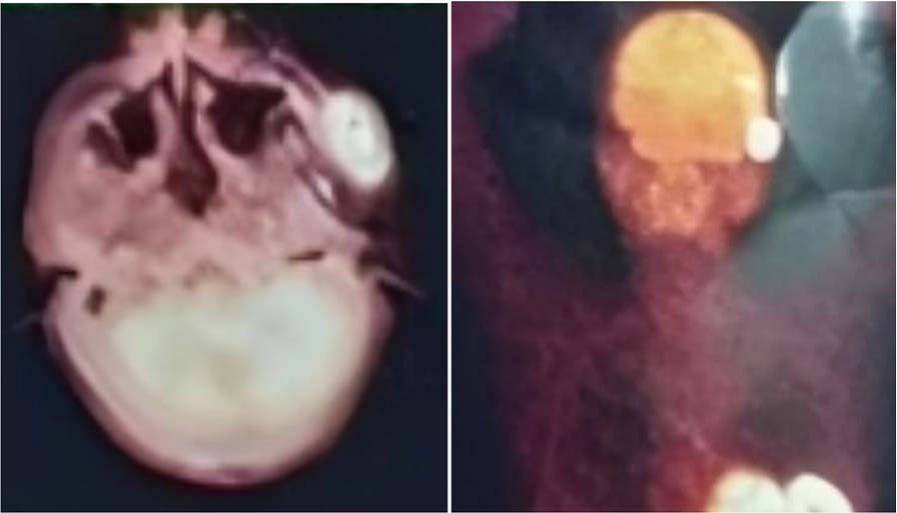
PET-CT demonstrating an unclear, heterogeneous mass that leachates into the skin and adipose tissue underneath it and the left masticatory muscle measuring 40 × 20 mm showing pathological metabolic activity.

A 57-year-old male was referred to the urology department with hematuria 6 months before. Computed tomographic (CT) showed a mass in the bladder without any other significant results (Fig. 1). Endoscopic examination showed a solid lesion within a large diverticulum where biopsies were performed and the histopathology examination showed UC (invaded the lamina propria, pT1). A radical cystectomy with ileal conduit was performed with extended pelvic lymph node dissection. The histological study indicated the presence of a high-grade UC, which invaded the muscularis propria to the perivesical fat (Fig. 2) with neural and lymphovascular invasion (Fig. 3), but the metastases to pelvic or obturator lymph nodes were absent. After that, he received adjuvant chemotherapy (Paclitaxel), but he could not tolerate the side effects of the drug, which led to the cessation of treatment after only two doses. Two months later, he complained of swelling on the left cheek (Fig. 4) with pain in the left shoulder. Positron emission tomography and CT (PET-CT) showed an unclear, heterogeneous mass that leaches into the skin and adipose tissue underneath it and the left masticatory muscle measuring 40 × 20 mm showing pathological metabolic activity (Fig. 5). In addition to the presence of osteolysis in the posterior arch of the left eighth rib with the formation of a tissue mass extending over 75 × 35 mm showing pathological metabolic activity. Surgical intervention was performed to remove the described masses, and the histological examination demonstrated the presence of UC. Then, he received chemotherapy (Paclitaxel, 6 doses), and unfortunately PET-CT showed a relapse which occurred 4 months later, where the eighth rib was removed and supraclavicular region. Then, he underwent immunotherapy (Nivolumab) and radiation therapy until his death 10 months after the first appearance of cheek metastasis. There was no recurrence in place of the removed mass on the cheek.

## Discussion

UC is the most common histological type of bladder cancer (~97% of malignant tumors of the bladder) [8]. The most common sites of metastases from UC are lungs, liver, brain, and bones. In the head and neck region, sites of metastases localization include the supraclavicular nodes and the skull [8]. The metastatic colonization of the cheek is exceedingly rare. Gross appearance of cutaneous metastases is not distinctive and may mimic many common dermatologic disorders [9].

The treatment for choice for metastatic bladder cancer is either chemotherapy, with the combination of gemcitabine and cisplatin or the methotrexate, vinblastine, doxorubicin, and cisplatin (MVAC) scheme; or palliative care [10]. Symptomatic patients may benefit from surgical resection of metastases in terms of tumor-related symptoms and performance status [11]. Local radiation therapy has also been reported to resolve cutaneous lesions, which did not respond to chemotherapy [12].

This case is characterized by the fact that the initial tumor was removed before the appearance of metastases and that surgical management of the cheek metastasis with subsequent chemotherapy was sufficient to prevent relapse of the metastasis site (up to a 10 months of follow-up) with no limitation in the patient’s oral movement. At the same time, the management of metastasis to the eighth rib in the same way (surgical removal with chemotherapy) did not prevent the occurrence of relapse of the excision site (although the excision edges were free of tumor) with metastases to new places.

For all of the above, the management of isolated metastases after radical cystectomy surgically remains controversial.

## Conclusions

Cutaneous metastasis of bladder carcinoma is extremely rare. An awareness of this rare clinical entity and high index of suspicion is needed for diagnosis. It is also a sign of poor prognosis, indicating a low survival rate and widespread disease.
